# Cyanids of Zinc in Facial Neuralgia

**Published:** 1878-01

**Authors:** 


### CYANIDS OF ZINC IN FACIAL NEURALGIA.

Dr. Luton, of Rheims, states that he has obtained excellent results from the cyanide of zinc in rheumatic facial neuralgia simulating cerebral rheumatism. He relates two cases in which, with intense facial neuralgia, there was continued and ardent fever, cephalagia and tenderness, on pressure at the points where the nerves emerged. The symptons rapidly abated under the use of the following mixture:—Cyanide of zinc one-fifth of a part, distilled cherry-laurel water twenty-five parts, and tragacanth mucilage mixture 100 parts. A tablespoonful, from hour to hour.

